# Underlying Sources of Response-Response Contingency Learning

**DOI:** 10.5334/joc.491

**Published:** 2026-02-19

**Authors:** Klaus Rothermund, Anna Martini, Philipp Sprengholz, Birte Moeller

**Affiliations:** 1 Friedrich Schiller University Jena, Jena, Germany; 2 Otto Friedrich University Bamberg, Bamberg, Germany; 3 Universität Trier, Trier, Germany

**Keywords:** Contingency learning, action sequences, serial reaction time task (SRTT), episodic response retrieval, response-response binding, law of recency, contingency awareness, propositional knowledge, implicit learning, habits

## Abstract

We examined contingency learning of response sequences (response-response contingency learning, RR-CL) in a serial reaction time task (SRTT), in which keys were pressed corresponding to the spatial position of dots appearing successively on a screen. First-order contingencies between pairs of successive responses were introduced into the task by biasing the transition probabilities between the spatial positions of subsequently appearing dots, rendering specific response sequences more likely than others. A pre-registered study (n = 40) revealed evidence for robust RR-CL effects, indicated by shorter (longer) response times for responses that were preceded by a likely (unlikely) preceding response. Part of this RR-CL effect was due to episodic retrieval of the most recent response sequence that started with the same response as the current sequence. Yet, a robust genuine RR-CL effect remained after controlling for recency-based episodic retrieval. This residual RR-CL effect was dependent on contingency awareness, with stronger residual RR-CL effects for sequences that were correctly identified as high or low in frequency, indicating that learning of response sequence contingencies partly reflects propositional knowledge. A reliable residual RR-CL effect, however, was obtained also in the absence of contingency awareness, indicating that another part of the learning of response sequence contingencies operates automatically and outside of awareness.

The aim of the present study was to investigate the learning of response sequences, and to identify its underlying processes. Response sequence learning can be understood as a form of response-response contingency learning (RR-CL), and thus is structurally similar to other forms of contingency learning, particularly stimulus-response contingency learning that have already attracted a lot of attention (SR-CL; Miller, 1987; Schmidt et al., 2007; for a recent review, see Schmidt, 2021). Although similar in principle, RR-CL and SR-CL also differ in important respects: In RR-CL, contingencies reflect dependencies between responses in successive trials, with a specific response R_x_ in trial n predicting the likelihood of a specific response R_y_ in trial n+1. In SR-CL, however, contingencies reflect dependencies between stimuli and responses that occur within trials. For instance, in the word-color CL paradigm, participants have to respond to the color in which words are presented. Words are irrelevant for the task but are predictive of the color in which they are presented, that is, each word is most often presented in a specific color, and less frequently in other colors (Schmidt et al., 2007). Thus, contingencies in SR-CL concern only individual responses that could also be learned via random response sequences, while RR-CL relies on regularities in response sequences. Another important difference concerns the task relevance of the contingent stimuli and responses. While responses in RR-CL are always task relevant, this is typically not the case for the stimuli that are investigated in SR-CL paradigms.

Extending research on contingency learning to the learning of response sequences is interesting for multiple reasons. First, it is interesting to see whether learning of contingencies which has already been demonstrated for various forms of SR relations (e.g., word-color contingencies, Schmidt et al., 2007, nonword-valence relations, Giesen, Rudolph, et al., 2025) can also be demonstrated for contingencies regarding the relation between successive responses. Second, response relations might be an interesting field of study, because it links contingency learning to research in implicit sequence learning (Nissen & Bullemer, 1987; see discussion below). Finally, it is interesting to see whether the processes that contribute to contingency learning of response sequences are similar or different from the processes that have been identified as underlying sources of SR contingency learning (e.g., Giesen et al., 2020, Giesen, Rudolph, et al., 2025; Rudolph & Rothermund, 2025; Rudolph et al., 2025a; Schmidt et al., 2020).

**The role of episodic retrieval of the last instance**. Drawing an analogy between RR-CL and SR-CL is interesting because recent research on SR-CL has identified an important factor contributing to SR-CL effects that might also play a role in RR-CL. Specifically, it has been shown that a large part of the SR-CL effect can be explained in terms of a simple retrieval of the last episode during which the current word was presented (the “law of recency”; Giesen et al., 2020; Schmidt et al., 2020). Since the last occurrence of each word is most likely a high frequency episode (i.e., an episode in which the word was presented in its typical color), retrieval of this episode will activate the response that is linked to this color, which will facilitate responding on a current high frequency trial, due to a match between the response that was given in the previous episode and the response that is required in the current trial. At the same time, episodic retrieval will delay responding on low frequency trials, since the retrieved response from the last episode (again, typically a high frequency episode) conflicts with the response that is required in the current trial.

Various studies demonstrated the influence of episodic response retrieval from the last occurrence, which explains a large share of the SR-CL effect (e.g., Giesen et al., 2020; Giesen, Duderstadt, et al., 2025; Giesen, Rudolph, et al., 2025; Güldenpenning et al., 2024; Rothermund et al., 2025; Rudolph & Rothermund, 2025; Rudolph et al., 2025a; Schmidt et al., 2020; Xu & Mordkoff, 2020). These episodic retrieval effects, however, do not constitute learning proper, they reflect a simple mechanism of copying the behavior that was shown during the very last situation in which the current stimulus appeared. Retrieval of a single episode does not meet the definition of learning, according to which learning consists in behavior adapting to environmental regularities (De Houwer & Hughes, 2020). Regularities, however, are defined in terms of relative frequencies or probabilities that have no meaning when applied to a single event. Furthermore, episodic retrieval of the very last episode that matches the current situation has no lasting effect on behavior, since encountering the next episode can completely reverse the SR-CL effect, leaving no enduring traces of learning (see Rudolph & Rothermund, 2026).

Importantly, the same rationale can also be applied to the learning of response sequences. In our study, we thus transferred the idea of episodic retrieval of the most recent episode to the learning of response sequences (RR-CL; see Figure 1). If, in a response sequence learning paradigm, R_x_ is most often followed by R_y_, then responding with R_y_ after having responded with R_x_ may reflect a simple retrieval of the last response sequence that started with R_x_. Since in most cases, R_x_ was followed by R_y_ during the last occurrence of R_x_, retrieving the last sequence starting with R_x_ will typically activate R_y_, and will thus lead to a facilitation of R_y_ in the current trial, and will interfere with a different, less likely response R_z_. In the context of RR-CL (i.e., sequences of two successive responses), an “episode” thus always refers to a pair of trials. Retrieving the last matching “episode” indicates that the first response of the current pair of successive responses retrieves the last pair of successive responses that started with the same response as the current pair. Importantly, pairs are not considered as separate, non-overlapping entities; instead, each trial (n) functions as the second element of a pair of trials that started with the preceding trial (pair [n-1,n]), and is simultaneously the first element of the pair that is constituted by the current trial and the immediately following trial (pair [n,n+1]).

**Figure 1:**
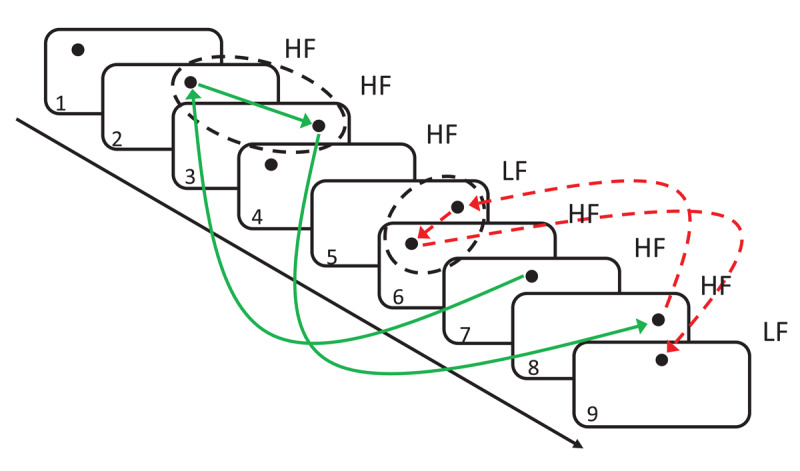
Illustration of recency-based retrieval of response sequences. *Note*. The green solid arrows illustrate the path that leads to a retrieval of a matching response: The “middle” response in trial 7 retrieves the last occurrence of the same response (trial 2). The response that was bound to the middle response in this sequence (i.e., a “right” response executed in trial 3) is then retrieved and influences responding in the following trial of the series (trial 8). In this case, a matching response was retrieved and reactivated (trial 8 also needs a “right” response), leading to facilitation. The red dotted arrows illustrate a situation in which a mismatching response is retrieved: Trial 8 will retrieve the last matching sequence starting with the same response (trials 5–6), and will reactivate the second element of the response sequence (trial 6) for the next upcoming trial (trial 9). In this case, a “left” response is retrieved from trial 6, but in trial 9 a “middle” response is to be given. In general, retrieval of matching (mismatching) responses is much more likely for high (low) frequency (HF/LF) trials, because the retrieved sequence is most likely a high frequency sequence.

Similar to what has been argued for SR-CL above, a RR-CL effect that reflects episodic retrieval of the last action sequence that started with the same response as the current sequence would not constitute learning proper for RR sequences. Effects of episodic retrieval of response sequences thus have to be taken into account when investigating RR-CL. Episodic retrieval has been shown to be a powerful mechanism in action control (for reviews, see Frings et al., 2020, 2024), both for SR episodes (e.g., Frings et al., 2007; Giesen & Rothermund, 2014, 2016; Hommel, 1998; Mayr & Buchner, 2006; Rothermund et al., 2005) as well as for RR sequences (e.g., Moeller & Frings, 2019a, b, 2021). As argued above, a simple and direct retrieval of the last matching episode does not meet the criteria of learning proper. Thus, episodic retrieval of the last matching response sequence has to be controlled for in order to determine effects of genuine RR-CL (for a similar rationale in SR-CL, see, Giesen, Duderstadt, et al., 2025; Giesen, Rudolph, et al., 2025; Giesen et al., 2020; Rothermund et al., 2025; Rudolph & Rothermund, 2025; Rudolph et al., 2025a; Schmidt et al., 2020).

**Sources of genuine contingency learning**. In studies investigating SR-CL, it has been shown that a small but robust residual SR-CL effect remains after controlling for or eliminating the influence of episodic retrieval (Rothermund et al., 2025; Rudolph & Rothermund, 2025; Rudolph et al., 2025a; Xu & Mordkoff, 2020). An interesting question then regards the underlying sources of this residual effect. In SR-CL, recent studies revealed that contingency awareness fully accounted for the residual SR-CL effect, indicating that the residual SR-CL effect reflects the operation of propositional knowledge rather than association formation (Giesen, Duderstadt, et al., 2025; Giesen, Rudolph, et al., 2025; Rothermund et al., 2025; Rudolph & Rothermund, 2025; Rudolph et al., 2025a). A second goal of our study thus was to investigate, whether – if a robust residual RR-CL effects is obtained after controlling for recency-based retrieval – such an effect depends on contingency awareness. If robust residual RR-CL effects can only be found for sequences for which participants can identify the contingency correctly, this would suggest a similar explanation of these effects in terms of propositional knowledge. If, however, robust residual RR-CL effects are obtained also in the absence of contingency awareness, then we would conclude that these genuine RR-CL effects reflect the operation of genuine associative learning, independent of participants becoming aware of the contingencies.

## The present study

**Figure 2:**
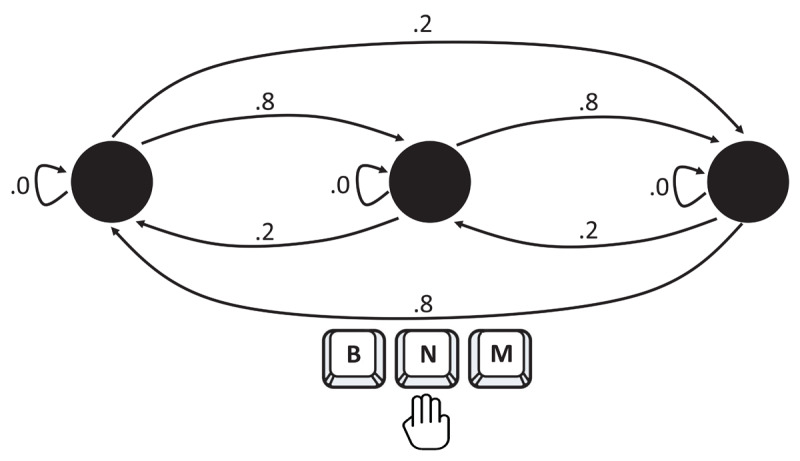
Transition probabilities between responses in the present study. *Note*. Response-response contingencies were created by biased transition probabilities between three spatial positions at which a dot could appear on the screen, indicating which key had to be pressed on a keyboard (left [B], middle [N], right [M]).

A simple variant of a serial reaction time task (SRTT; Nissen & Bullemer, 1987) was used to investigate contingency learning for response sequences (RR-CL). Specifically, in each trial of the task, a dot appeared at one of three possible locations on the screen (left, middle, or right) and a corresponding key had to be pressed on the keyboard. Transition probabilities between responses were biased, rendering some response sequences highly likely, and others unlikely; for instance, after having pressed the middle key in trial n-1, the dot would appear in the right (left) location with a probability of .8 (.2) in trial n (see Figure 2).

RR-CL effects were assessed by comparing RTs for high and low frequency response transitions. Importantly, the influence of episodic retrieval processes was estimated by coding for each trial whether the most recent sequence that started with the same initial response was followed by either the same or a different response compared to the current sequence (Figure 1). Statistically controlling for the effect of response retrieval yielded a residual RR-CL effect that reflects genuine response sequence learning. At the end of the experiment, we also assessed contingency awareness (CA) by letting participants categorize the likelihood of each possible response sequence. This allowed us to investigate the dependency of residual RR-CL effects on CA.

## Method

### Ethics approval, pre-registration, and open access

Ethical approval was granted by the Ethics Committee of the FSU Jena (FSV 20/005). Prior to data collection, the method, design, hypotheses, data preparation, and planned analyses were pre-registered online (https://aspredicted.org/g36d-krnx.pdf). All data and analyses scripts are available at https://osf.io/hp8rm/?view_only=3de01cc02eb9427fba05738268d756ee. The experiment can be run via https://sprengholz.github.io/actionsequences-v2/, the program code can be accessed via https://github.com/sprengholz/actionsequences-v2.

### Required sample size and a-priori power calculations

Based on a pilot study in which a large residual effect of RR-CL was obtained after controlling for episodic retrieval of the last response sequence, we aimed at collecting data from a sample of n = 40 participants, which would allow us to detect a medium sized effect for residual RR-CL (d = .5) with a power larger than .9.

**Participants**. 40 participants were recruited online from Prolific Academic (24 female, 16 male; *M*_age_ = 24.0 years). All participants were pre-screened to be aged between 18 and 30 years, having German as their mother tongue, and using a notebook or desktop computer. The experiment had an average duration of less than 15 minutes and participants were compensated with £2.00 for taking part. All participants gave informed consent prior to taking part in the study.

**Design**. The experiment had a 2 (contingency: high vs. low frequency response sequence in the current trial) × 2 (last occurrence: same vs. different second response during the last response sequence that had started with the same initial response as the current one) repeated measures design, with all factors being manipulated within participants. Reaction times (RT) served as the dependent variable of interest.

**Materials and procedure**. The online experiment was implemented with jsPsych (de Leeuw, 2015). At the start of each experiment, demographic information (gender, age, handedness) was collected, followed by the consent page. If participants consented to take part, instructions followed; otherwise, the study was terminated.

Participants’ task was to identify the position of a black dot appearing in the left, middle or right part of a white screen by pressing one of three keys (‘B’, ‘N’, or ‘M’) on the keyboard. Each participant received 900 successive trials of the task. After completing blocks of 300 trials participants could take a short break. At the beginning of each block, participants practiced the task for 10 trials. The trial sequence started with an arbitrary position of the dot in the first trial, and then continued by choosing the next position depending on the contingencies that were implemented. The same position could never be repeated in the next trial. For each of the three positions, one of the two remaining positions was defined to be the high probability successor, and was chosen with a probability of .8 in the next trial, whereas the other position was the low probability successor, and was presented with a probability of .2 in the next trial^1^ (Figure 2). Two possible sets of contingencies were defined (set 1: left-middle/middle-right/right-left as high probability sequences, and left-right/middle-left/right-middle as low probability sequences; set 2: left-right/middle-left/right-middle as high probability sequences, and left-middle/middle-right/right-left as low probability sequences) that were counterbalanced across participants. For each participant, the same contingencies between responses were realized throughout the entire experiment. Note that the sequential structure that was implemented in our study was purely probabilistic, not deterministic, in that we did not control for the absolute frequency of pairs of successive responses, nor did we control the order in which the pairs of trials were presented. Instead, each trial initiated an independent random process during which the position of the dot was selected based on the dot position of the preceding trial (with a probability of zero for the same position, a probability of .8 for the highly likely next position, and a probability of .2 for the less likely next position). Given the large number of trials, this procedure ended up with producing relative frequencies of successive pairs of responses that were very close to the probabilities that were used to implement the contingencies. Table A1 gives the total number of trials for each of the four cells of our design (contingency × last occurrence).

Each trial of the color classification task started with the presentation of a black dot in one of the three locations, which stayed on the screen until a response key was pressed. Afterwards, the next dot immediately appeared on the screen. Inaccurate responses elicited a feedback message (“*Falsche Taste! Dr*ü*cke B f*ü*r einen links, N f*ü*r einen mittig und M f*ü*r einen rechts gezeigten schwarzen Punkt.”* [Wrong key! Press B for a left dot, N for a center dot, and M for a right dot]). Feedback was displayed at the top of the screen in white font on red background. After the 300^th^ and 600^th^ trial of the task, participants were asked to take a short break, the duration of which they could determine for themselves, and to continue working on the task by pressing the space bar.

**Figure 3:**
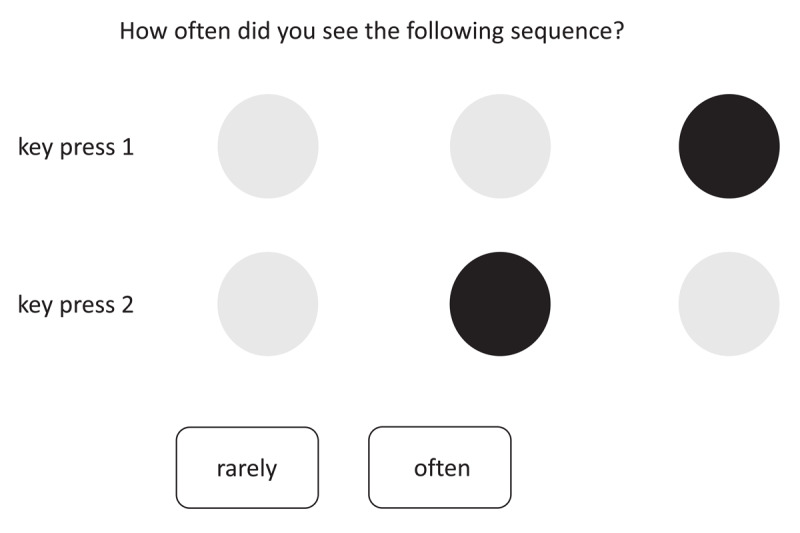
Contingency awareness assessment at the end of the experiment for a sample sequence.

After completing all trials of the task, contingency awareness was assessed by having participants classify each possible response sequence as being either highly probable or improbable. Each of the six possible sequences of two successive dot positions and corresponding key presses was presented visually on the screen by displaying two visual displays of possible dot positions below each other (see Figure 3). Participants then had to indicate whether this sequence occurred often or only rarely during the experiment. On average, 62.9% of these classifications were correct, which indicates contingency awareness that is significantly above chance (50%), *t*(40) = 3.92, *p* < .001, but is far from being perfect.

### Data preparation and analysis plan

Prior to analyses, trials in which an error occurred in the current sequence (3.9%), as well as trials that were either the first pair of trials that started with this response in the current block of trials (0.1%), or where an error was made in the preceding pair of trials that started with the same response (8.0%) were discarded. Also, responses faster than 100 ms or slower than 1.5 interquartile ranges above the 75^th^ percentile of the individual RT distribution were regarded as RT outliers (Tukey, 1977) and were also excluded (2.7%). This resulted in a total of 30,667 valid RTs that entered into the analyses (M = 419 ms, SD = 91 ms, range = [102 ms, 844 ms], skewness = .60, kurtosis = .47).

After errors and outliers had been eliminated, data were analyzed with hierarchical multi-level regression analyses, treating trials as nested within subjects, while allowing for random intercepts to control for differences in response speed between participants. RT was the dependent variable of interest. Each trial was coded according to whether (a) the current response was a highly frequent vs. infrequent successor of the previous response (CL: hf vs. lf), (b) whether the last but one sequence was a high or low frequency sequence (PCL),^2^ (c) whether the last sequence that started with the same response as the current one either had the same or a different second response as the current sequence; that is, episodic retrieval (ER: same vs. different response), and (d) whether the frequency of occurrence had been correctly identified for the current response sequence during the contingency awareness assessment at the end of the experiment (CA: correct vs. incorrect). All predictors indicated a contrast between two conditions and were coded to have (1) a mean of zero across all trials within the analysis, and (2) a difference of 1 between the two weights (see Rudolph & Rothermund, 2025).^3^ Thus, the resulting regression coefficients reflect the difference between the two conditions (in milliseconds), and main effects and interactions of the predictors can be interpreted simultaneously due to the fact that the centered predictors and their products (interactions) are orthogonal. The predictor variables and their interactions were entered in a stepwise fashion into the regression equation to see how introducing additional predictors (e.g., episodic retrieval) changes the effect of the CL effect.

## Results

In a hierarchical linear regression, RT for individual trials, nested within participants, was predicted by various factors (see Table 1), coding contingency of the current (CL) and the immediately preceding response sequence (PCL), episodic retrieval of a matching vs. mismatching response from the last sequence that started with the same response as the current one (ER), and correct identification of the frequency of the current sequence (CA). In a first step, CL, PCL, and their interaction were entered as predictors in the analysis. This yielded a large and significant RR-CL effect: On average, participants responded 65 ms faster in high contingency compared to low contingency sequences, *t*(30,630) = –58.41, *p* < .001. In addition, responses were 17 ms faster if the previous sequence had been a high contingency sequence, *t*(30,630) = –16.37, *p* < .001. Furthermore, responses in reversal sequences were slower compared to responses in non-reversal sequences, as indicated by a significant CL × PCL interaction, *t*(30,630) = –11.16, *p* < .001. In a second step, episodic retrieval (ER) was entered as an additional predictor. Entering ER produced a robust effect for recency-based response retrieval with responses being 56 ms faster for trials that required the same response as during the last occurrence compared to trials in which a different response had to be given as during the last appearance of the word, *t*(30,631) = –51.79, *p* < .001. Entering ER into the regression equation led to a reduction in the strength of the CL effect, which, however, was still very strong (residual RR-CL effect: *M* = –32 ms, *t*[39,631] = –25.51, *p* < .001). In a final third step, contingency awareness (CA) and its interaction with CL, as well as the ER × CA interaction were entered as additional predictors into the model. The CL × CA interaction was significant (*M* = –24 ms, *t*[30,645] = –8.96, *p* < .001), indicating that the residual CL effect was modulated by contingency awareness. The ER × CA interaction missed significance, *t*(30,634) = 1.81, *p* = .07, indicating that the strength of ER is independent from contingency awareness, which corresponds to what has previously been found for SR contingency learning (Rudolph et al., 2025b).

**Table 1:** Results of a stepwise multi-level regression analysis predicting RT based on contingencies for the current and previous sequence (CL and PCL, step 1), episodic retrieval (ER, step 2), contingency awareness (CA) and its interactions with CL and ER (step 3).


PREDICTOR	MODEL 1	MODEL 2	MODEL 3

Intercept	418*** [403.1, 432.0]	418*** [403.3, 431.9]	419*** [404.1, 432.9]

CL (hc vs. lc)	–65*** [–67.6, –63.2]	–32*** [–34.4, –29.5]	–33*** [–35.9, –30.7]

PCL (hc vs. lc)	–17*** [–19.5, –15.3]	–23*** [–25.2, –21.2]	–23*** [–25.1, –21.1]

CL × PCL	–31*** [–36.0, –25.3]	–51*** [–56.6, –46.1]	–52*** [–56.9, –46.4]

ER (matching vs mismatching)		–56*** [–57.7, –53.5]	–56*** [–57.8, –53.6]

CA (correct vs. incorrect)			–9*** [–10.9, –6.8]

CL × CA			–24*** [–29.5, –18.9]

ER × CA			4 [–0.4, 8.8]

BIC	352091	349518	349371

∆ BIC	–	–2573	–147


*Note*. *p < 0.05, **p < 0.01, ***p < 0.001. CL/PCL, contingency in the current or preceding response sequence; hc/lc: responses following the previous response with high/low probability. The interaction of CL and PCL indicates differences between reversal and non-reversal sequences. ER, episodic retrieval; matching/mismatching second response during the last occurrence of a sequence that started with the same response; CA, contingency awareness; correct/incorrect: identification of the likelihood of occurrence for the current response sequence. BIC, Bayesian information criterion. We implemented a person specific intercept to control for individual differences in RTs. All other variables were implemented on a trial level. Values in brackets indicate the 95% confidence interval (lower and upper limit) for each regression weight. Regression weights (*ß*) reflect the difference in milliseconds between the conditions that define a contrast.

Following up on the CL × CA interaction, we analyzed the strength of the residual RR-CL effect separately for each awareness condition. For sequences for which the frequency of occurrence had been correctly identified (CA+), a residual RR-CL effect of 40 ms emerged (*t*[22,022] = –23.85, *p* < .001). For sequences without contingency awareness (CA-), a significant residual RR-CL effect of 19 ms was found, that was half as large as for the CA+ trials (*t*[8,610] = –8.50, *p* < .001).

An analysis of accuracy data revealed a similar pattern of results (see Table A2 in the Appendix). We also found a significant effect for RR-CL, that was reduced – but not eliminated – by introducing ER. Different from the results of the RT data, however, we did not find a modulation of CL effects by CA for the accuracy data.

## Discussion

In the present study, our primary goal was to investigate contingency learning of response-response contingencies, while controlling for the influence of episodic retrieval. A simple serial reaction time task (SRTT) was used in which response-response contingencies were implemented by using biased response transition probabilities. A large RR-CL effect emerged in this task that was reduced to half its original size after controlling for episodic response retrieval driven by the last response sequence starting with the same response.

This finding demonstrates that – similar to what was observed in SR-CL (e.g., Giesen et al., 2020; Giesen, Duderstadt, et al., 2025; Giesen, Rudolph, et al., 2025; Güldenpenning et al., 2024; Rothermund et al., 2025; Rudolph & Rothermund, 2025; Rudolph et al., 2025a; Schmidt et al., 2020; Xu & Mordkoff, 2020), a large part of the RR-CL effect is driven by recency-based response retrieval of a single, most recent episode (note that in the case of RR sequences, the most recent “episode” refers to the pair of successive trials that started with the same response as the current pair of trials, see above). This also suggests that some of the existing evidence for sequence learning without awareness may actually rely on episodic retrieval effects.

Similar to what we found for SR-CL (Giesen, Duderstadt, et al., 2025; Giesen, Rudolph, et al., 2025; Rothermund et al., 2025; Rudolph & Rothermund, 2025; Rudolph et al., 2025a), residual RR-CL effects were larger if the contingencies for the respective response sequences could be identified correctly, indicating that part of the genuine RR-CL effect reflects propositional beliefs and propositional learning (De Houwer, 2014; Mitchell et al., 2009). Participants might realize the dependency between successive presentations of the dots, and form beliefs like “Most of the time, after a dot has appeared in the middle of the screen, the next dot will appear on the left side of the screen. I thus should expect the next dot on the left, and be prepared for a ‘left’ response.” If correct, such a belief will lead to faster responses for high contingency trials, that is, for trials in which the dot appears at the more probable location, but will interfere with responding if the dot appears at the less likely spatial position. Our other findings, however, show that RR-CL is governed by different principles than is SR-CL.

First, the proportion of the RR-CL effect that reflects genuine RR-CL (i.e., the residual CL effect after controlling for ER) is much larger than what was typically observed in SR-CL. Note that SR-CL was in some cases entirely absent after correcting for episodic response retrieval (Giesen et al., 2020; Schmidt et al., 2020). At the very least this finding reflects much faster learning progress for response-response than for stimulus-response contingencies. Second, and even more importantly, although the residual RR-CL effect still depended on CA, a highly robust residual RR-CL effect also emerged for those sequences for which the contingencies were not identified correctly. Together these findings suggest that recency-based episodic retrieval is likely to affect learning patterns in SRT tasks but cannot account for all sequence learning. In particular, retrieval of the last sequence that started with the same response does not fully account for RR-CL effect that were obtained in the absence of contingency awareness.

The finding of RR-CL without CA in particular distinguishes RR-CL from SR-CL, where genuine SR-CL effects were repeatedly shown to be completely dependent on contingency awareness (Giesen, Duderstadt, et al., 2025; Giesen, Rudolph, et al., 2025; Rothermund et al., 2025; Rudolph & Rothermund, 2025; Rudolph et al., 2025a). In theoretical terms, such an effect of genuine RR-CL without contingency awareness suggests that there is another source of RR-CL that is unrelated to conscious insight into the contingencies and to the formation of propositional beliefs. Promising candidates to explain these residual effects in the absence of contingency awareness are either cumulative memory models (e.g., Barth et al., 2019; Jamieson et al., 2012), models of association formation in the tradition of the Rescorla-Wagner model (Rescorla & Wagner, 1972), or race models of episodic memory, in which episodes with higher frequency of occurrence have a higher chance to win the race (Logan, 1988).

With the current data, we cannot distinguish between these different accounts. Importantly, however, the mere existence of genuine RR-CL effects that are independent of contingency awareness further attests to the claim that at least part of the RR-CL effect operates in an automatic and goal-independent fashion (cf. Balleine & Dezfouli, 2019; Jiménez et al., 1996; Moeller & Pfister, 2022; Nissen & Bullemer, 1987), since this is a common feature of all three models. This finding of genuine contingency learning effects without contingency awareness sets RR-CL apart from SR-CL, for which at least the currently available evidence suggests that genuine SR-CL effects are completely dependent on contingency awareness and thus can be assumed to operate on a conscious level (Giesen, Duderstadt, et al., 2025; Giesen, Rudolph, et al., 2025; Rothermund et al., 2025; Rudolph & Rothermund, 2025; Rudolph et al., 2025a).

*Implications for implicit sequence learning*. The findings of our study on response-response contingency learning also relate to a large literature of implicit sequence learning (Nissen & Bullemer, 1987) and habit formation (Dezfouli & Balleine, 2013). The standard paradigm to investigate implicit sequence learning is the serial response time task (SRTT, Nissen & Bullemer, 1987). In this paradigm, transitions between different responses typically follow certain rules (denoted as the “grammar” of the task), and responses are faster and more accurate for blocks of trials that are in accordance with the rules of the task compared to blocks of trials in which no rules apply (random sequences). RR-CL can be considered a very simple form of implicit sequence learning in which responding on likely response sequences is compared to response sequences which have a low probability.

Our study thus is potentially relevant for the literature on implicit sequence learning. On a general note, the reported findings further support the claim that regularities in response sequences influence behavior, in that responding to more likely sequences is facilitated compared to less likely sequences.

In more detail, our study shows that episodic retrieval processes play an important role in RR-CL, and thus should also be considered as a potential factor that might influence implicit sequence learning. This point is important for two major reasons: First, effects that are based on episodic retrieval do not reflect learning proper (see above) since they reflect the influence of single instances rather than regularities (De Houwer & Hughes, 2020), and do not have a lasting influence on performance (e.g., Rudolph & Rothermund, 2026). Insofar effects of implicit sequence learning are due to episodic retrieval, they should also not be considered as reflecting learning proper.

Second, processes of episodic retrieval typically operate automatically and independently of awareness. If episodic retrieval also influences implicit sequence learning, these influences might feign implicit learning processes, even if the genuine learning process (after controlling for episodic retrieval) is not automatic (see, e.g., Giesen, Rudolph, et al., 2025; Rudolph et al., 2025a; Rudolph & Rothermund, 2025). This is particularly important given the extensive debates whether regularities in the sequence of actions can be learned without participants becoming aware of these regularities (e.g., Jiménez et al., 1996; Nissen & Bullemer, 1987), and – once acquired – influence behavior in a way that is independent of an individual’s goals (Balleine & Dezfouli, 2019). The literature regarding the question of automaticity and awareness in the acquisition and operation of response sequences is tremendously rich and diverse (e.g., see reviews by Abrahamse et al., 2010; Keele et al., 2003; Schwarb & Schumacher, 2012; Timmermans & Cleeremans, 2015). Yet, the influence of episodic retrieval processes as specified by the law of recency (Giesen et al., 2020) has been neglected in the literature on implicit sequence learning.^4^ Our findings show, however, that this might be an important contributor to these effects.

For researchers investigating response sequence learning it will thus be important in the future to control for effects of recency-based retrieval, in order to be able to identify effects that reflect genuine learning of RR sequences, and whether these genuine effects are automatic in nature or not. Our findings suggest that a robust effect for genuine RR-CL can be demonstrated even after controlling for recency-based retrieval of the last response sequence that started with the same response. Furthermore, although contingency awareness modulated the strength of genuine RR-CL effects, these effects were shown to operate also in the absence of contingency awareness, which supports the claim that part of the RR-CL effect can be attributed to automatic learning processes.

It should be noted that typical implicit sequence learning experiments use second-order contingencies (i.e., contingencies between pairs of responses that predict a successive response), and control for first-order response contingencies (single responses predicting the next response). In a second-order contingency situation, retrieval of response sequences based on a single starting response cannot explain the effect. Instead, these simple retrieval effects would always result in the retrieval of a mismatching response. The idea of episodic retrieval can, however, easily be transferred also to the more complex situation of second order contingencies, by assuming that triplets of successive responses are stored in episodic memory, so that pairs of successive response can also function as retrieval cues for these episodes, retrieving the final response of the triplet. To investigate the influence of these episodic retrieval effects on implicit sequence learning, probabilistic variants of a second order RR-CL paradigm are needed, which allow researchers to control for episodic retrieval, and to estimate genuine RR-CL effects after controlling for these retrieval effects.

### Limitations

Our study is the first to investigate genuine effects of response sequence learning (RR-CL) after controlling for effects of recency-based episodic retrieval of the last matching response sequence. When interpreting the results of our study, however, it has to be taken into account that the findings might in part reflect the specific manipulations and assessments we chose for our study.

First, we implemented a fairly simple first-order contingency of response sequences,^5^ using rather strong contingencies. Results might differ for studies in which the contingencies are weaker, and/or more complex (e.g., second order) contingencies between responses are investigated. Weaker or more complex contingencies might be more difficult to detect, resulting in reduced contingency awareness. Given that the present manipulation of contingencies already resulted in contingency awareness that was far from perfect, and robust genuine RR-CL effects still obtained even when contingency awareness was absent, we would expect robust RR-CL effects also for these manipulations.

Second, although our contingency manipulation resulted in large and highly robust CL effects, even after controlling for recency-based episodic retrieval effects, contingency awareness was still comparatively low. Although low contingency awareness is not unusual in studies investigating response sequence learning, a possible reason for low contingency awareness might also be that the measure of contingency awareness that we used might not have been perfectly reliable. Although we tried to present the sequences in a way that was highly similar to the situation that was presented during the experiment proper, there still were differences. Importantly, participants did not have to press keys during the assessment of contingency awareness for the sequences, which might have helped them to retrieve the knowledge about whether the sequences were typical or atypical.^6^ Alternatively, rather than reflecting about the likelihood of each possible sequence (categorizing it as either highly likely vs. less likely), participants might have formed knowledge regarding the relative frequency with which certain pairs of successive pairs of spatial positions or responses are presented in the experiment (e.g., “After having pressed the left key, it is much more likely that the next dot will appear in the middle than on the right side.”). Instead of asking participants to categorize the likelihood of occurrence separately for each of the six possible sequences, asking for relative judgments in which they had to compare the relative frequency of two pairs of sequences might have better captured the knowledge about contingencies (e.g., “Which of the following sequences was presented more often during the experiment left-middle or left-right?”).^7^

Imperfect reliability of the contingency awareness measure will lead to an underestimation of contingency awareness, and to an overestimation of the effect estimates for the unaware condition, due to some residual awareness still being present even for the incorrect responses in the contingency awareness tests (Timmermans & Cleeremans, 2015). The problem of residual awareness even for participants who cannot correctly identify contingencies (or stimuli) has been haunting the investigation of unconscious cognition for decades (e.g., Holender, 1986) and has also been a topic of debate in response sequence learning. Yet, it should also be mentioned that a task which aims at measuring awareness also affects the construction of consciousness (see Timmermans & Cleeremans, 2015), which could lead to an *over*estimation of awareness that was not present during the experiment. The mere fact that we did find a modulating effect of CA on the strength of the residual RR-CL effect already is positive evidence that our measure captures meaningful differences in the level of contingency awareness. It does not suffice, however, to exclude the possibility of residual awareness of contingencies at least in some cases when these contingencies were not identified correctly.

Another limitation of our study regards the perfect confounding of responses with the spatial location at which the dot appears (right position – right keypress, etc.). It is thus unclear, which kind of response sequences were actually learned in our study. It could be that participants learned sequences of keypresses, alternatively, they could also have learned to expect the next dot at a certain spatial position, which then could have led to learned sequences of eye movements (overt attention responses) or to learned sequences of shifting the focus of attention (covert attention responses). In order to disentangle these processes, future research would have to systematically manipulate keypresses and spatial positions independently. Importantly, however, since we always presented exactly the same stimulus at each of the three possible locations, we at least can rule out an explanation of our findings in terms of S-S learning, that is, by developing a perceptual readiness to identify the next stimulus based on an expectation of which features the next stimulus will have (see Koch & Hoffmann, 2000a, b).

## Conclusion

Response sequence learning effects are strongly influenced by retrieval of the last matching response sequence. Controlling for the influence of recency-based episodic retrieval revealed robust genuine RR-CL effects. These residual effects were modulated by CA, with larger RR-CL effects for contingencies that could be correctly identified, indicating that part of the genuine RR-CL effects is driven by propositional beliefs. A robust RR-CL effect, however, also obtained for response sequences for which contingencies could not be identified correctly. Especially the latter finding sets RR-CL apart from SR-CL, and demonstrates an influence of learning processes that operate automatically, independent of an individual’s beliefs and goals.

## Data Accessibility Statement

All data and analyses scripts are available at https://osf.io/hp8rm/?view_only=3de01cc02eb9427fba05738268d756ee.

## Footnotes

Implementing these biased transition probabilities corresponds to a contingency coefficient of ΔP = .8 – .2 = .6, which is the difference in conditional probabilities for a response in trial n given that it is preceded by either its likely or its unlikely predecessor in trial n-1. The entropy for the distribution of probabilities across the two possible responses in trial n conditional on the response in trial n-1 is H = .72 bits (lower than the entropy if both response options had an equal probability, H = 1 bit).

This factor was not pre-registered, but was introduced to control for effects of response-reversal sequences (e.g., Reed & Johnson, 1994; Vaquero et al., 2006), which was recommended by reviewer Dr. Luis Jiménez, whom we would like to thank for this suggestion. Specifically, the interaction between the contingency status of the current and the immediately preceding sequence of two responses indicates whether the resulting sequence of three responses is (or is not) a reversal sequence: In our paradigm, combinations of one high and one low contingency sequence always result in a reversal sequence, whereas two high frequency or two low frequency sequences in succession always result in a non-reversal sequence. For instance, if left-middle (l-m), middle-right (m-r), and right-left (r-l) are the high frequency sequences, then high-low (e.g., l-m-l) and low-high (e.g., m-l-m) combinations lead to reversals, whereas high-high (e.g., l-m-r) or low-low (e.g., m-l-r) combinations never do. Importantly, including this factor in the analyses does not change the pattern of results for the other factors.

The general formulas that will satisfy these standards are: (1) \[{W_1} = \frac{{{f_2}}}{{\left( {{f_1} + {f_2}} \right)}}\] , and (2) \[{W_2} = \frac{{ - {f_1}}}{{\left( {{f_1} + {f_2}} \right)}}\]
where *w*_1_ and *w*_2_ are the regression weights that define a contrast, and *f*_1_ and *f*_2_ are the number of trials per condition. Equal trial frequencies in each condition will result in a contrast coding of *w*_1_ = .5 and *w*_2_ = -.5. If the frequencies are unequal, however, this will result in unequal weights, so that the condition with the higher frequency receives a lower (absolute) weight (and vice versa).

Recent accounts of sequence learning have explained these effects in terms of memory processes (Jamieson et al., 2012; see also Barth et al., 2019). The underlying idea is that the current response sequence re-activates memory traces of previous response sequences that are similar (i.e., that started with a similar response). These memory-based accounts (e.g., adaptations or extensions of the MINERVA 2 architecture, Hintzman, 1984) model the cumulative influence of all experiences, so that each previously encountered instance that shares some features with the current episode contributes to the “echo” that is elicited by the current response sequence. The “echo” thus can be seen as an accumulation of previous experiences. With more and more episodes contributing to the memory base, the more stable the influence of contingencies on responding becomes, nicely mimicking an asymptotic learning curve. Memory models are very powerful and flexible, they can be configured in different ways, assigning different weights to previous experiences depending on the temporal distance of when the event was encountered. Parameters of a memory model thus can also be configured in such a way that only the most recent episode gets a positive weight, whereas all previous episodes receive a weight of zero, thus restricting the influence of memory to just the most recent encounter of the stimulus, which essentially models the influence of pure recency-based episodic retrieval. Importantly, however, and as we argued above, such a parametrization would no longer reflect genuine learning, since (a) memory effects would no longer reflect regularities, which can only be defined in terms of relative frequencies or probabilities, which cannot be defined for a single instance, and (b) any episode would influence only the immediately following episode in which a similar situation is encountered, and would not have a lasting effect on behavior (or memory), which is another pre-requisite for labeling a phenomenon as learning (see De Houwer & Hughes, 2020).

First-order contingency here indicates a dependency between pairs of responses, that is, between a response and the immediately preceding response. Zero-order contingencies would reflect differences in response probabilities at the trial level, independent of all preceding trials (in our study, there was no zero-order contingency, since all three responses had the same overall probability of occurrence, *p* = 1/3). Second (or higher) order contingencies would reflect a dependency of the probability of a response in trial n on the response that had to be given in trial n-2 (or an earlier trial) after controlling for the influence of the trial n-1 response. In our study, no such influence was implemented, that is, trials n-2 and earlier had not influence on trial n over and above the influence of trial n-1.

We also tested a variant of assessing contingency awareness by inserting “guessing trials” in which participants had to guess the position of the next upcoming dot by pressing the corresponding key in a pilot study. With this procedure, however, contingency awareness was close to chance level. We thus decided to go with the explicit verbal assessment of contingency awareness for the present study, which is also more comparable to the form of contingency awareness assessment that was used in studies investigating SR-CL (e.g., Rudolph & Rothermund, 2025; Rudolph et al., 2025a).

We would like to thank Iring Koch for pointing out that this form of question might better capture knowledge about RR sequences.

## Appendix

**Table A1:** Number of experimental trials for each combination of the experimental design across participants.

	CONTINGENCY	

Last sequence with same first response	High	Low

Same second response	22,746	1,486

Different second response	5,738	5,669

*Note*. Each trial was coded as (a) high or low contingency depending on whether it was preceded by its likely or unlikely predecessor, and (b) as retrieving the same or a different response from the last sequence that had started with the same initial response as the current one.

**Table A2:** Results of a stepwise multi-level regression analysis predicting accuracy (1 = correct response, 0 = erroneous response) based on contingencies of the current or immediately preceding sequence (CL and PCL, step 1), episodic retrieval (ER, step 2), contingency awareness (CA) and its interactions with CL and ER (step 3).

PREDICTOR	MODEL 1	MODEL 2	MODEL 3

Intercept	.963*** [.955, .971]	.963*** [.955, .972]	.963*** [.955, .972]

CL (hc vs. lc)	.092*** [.087, .097]	.065*** [.059, .071]	.064*** [.058, .070]

PCL (hc vs. lc)	-.003 [-.008, .002]	.002 [-.003, .007]	.002 [-.003, .007]

CL × PCL	.033*** [.021, .046]	.048*** [.036, .061]	.049*** [.036, .062]

ER (matching vs mismatching)		.044*** [.038, .049]	.044*** [.038, .049]

CA (correct vs. incorrect)			.001 [-.004, .007]

CL × CA			.008 [-.004, .021]

ER × CA			-.021*** [-.033, -.010]

*Note*. **p* < 0.05, ***p* < 0.01, ****p* < 0.001. CL/PCL, contingency in the current and preceding response sequence; hc/lc: responses following the previous response with high/low probability. ER, episodic retrieval; matching/mismatching second response during the last occurrence of a sequence that started with the same response; CA, contingency awareness; correct/incorrect: identification of the likelihood of occurrence for the current response sequence. We implemented a person specific intercept to control for individual differences in accuracy rates. All other variables were implemented on a trial level. Values in brackets indicate the 95% confidence interval (lower and upper limit) for each regression weight. Regression weights (*ß*) reflect the difference in %correct responses between the conditions that define a contrast.
